# Activated Rho GTPases in Cancer—The Beginning of a New Paradigm

**DOI:** 10.3390/ijms19123949

**Published:** 2018-12-08

**Authors:** Pontus Aspenström

**Affiliations:** Rudbeck Laboratory, Department of Immunology, Genetics and Pathology (IGP), Uppsala University, SE 751 85 Uppsala, Sweden; pontus.aspenstrom@igp.uu.se

**Keywords:** Rho GTPases, atypical Rho GTPases, oncogenes, actin, stress fibers, cell migration

## Abstract

Involvement of Rho GTPases in cancer has been a matter of debate since the identification of the first members of this branch of the Ras superfamily of small GTPases. The Rho GTPases were ascribed important roles in the cell, although these were restricted to regulation of cytoskeletal dynamics, cell morphogenesis, and cell locomotion, with initially no clear indications of direct involvement in cancer progression. This paradigm has been challenged by numerous observations that Rho-regulated pathways are often dysregulated in cancers. More recently, identification of point mutants in the Rho GTPases Rac1, RhoA, and Cdc42 in human tumors has finally given rise to a new paradigm, and we can now state with confidence that Rho GTPases serve as oncogenes in several human cancers. This article provides an exposé of current knowledge of the roles of activated Rho GTPases in cancers.

## 1. Introduction

The Rho GTPases comprise a subfamily of the Ras superfamily of small GTPases. They include 20 members that can be further separated into the typical, or classical, Rho GTPases, and the atypical Rho GTPases [1,2]. Some of the Rho GTPases have a tissue-specific expression, e.g., Rac2 and RhoH in hematopoietic cells, but a majority of them have a ubiquitous expression pattern [1]. According to the original paradigm, the Rho GTPases have important roles in regulation of the organization of the actin cytoskeleton, although in contrast to Ras, they were not considered to be oncogenes [3]. This paradigm is now about to shift as there have been numerous demonstrations of clear links between the activity of Rho GTPases and oncogenesis. One such demonstration is that transcriptome analyses have shown that Rho-dependent signaling pathways are dysregulated in human cancers. Often the expression levels of regulatory proteins are altered, such that either there is overexpression or constitutive activation of their activating proteins, or there is suppression of expression of their negative regulators. Sometimes, specific members of the Rho GTPases are themselves overexpressed or under expressed in cancers. The consequence of this dysregulation of expression of Rho GTPases is the overactivity of Rho-dependent signaling pathways [3].

Recently, another mechanism for cancer-associated alterations has emerged: Activating mutations. To date, these mutations have predominantly been identified for the classical members of the Rho GTPases, Rac1, Rac2, RhoA, and Cdc42. These findings have thus challenged the concept of Rho GTPases not being oncogenes; in contrast, some members of this family of small GTPases should undoubtedly be considered as *bona-fide* oncogenes in certain tumor types. This review describes the historical background to the current view on cancer-related mutations in the Rho GTPases, and it also touches upon the atypical Rho GTPases; i.e., Rho proteins that are constitutively active in their wild-type form.

## 2. Identification of the Classical Rho GTPases

The identification and cloning of the Rho (Ras homologous) gene is a classic example of a serendipitous discovery. This discovery was made by Pascal Madaule in the laboratory of Richard Axel at the Institute of Cancer Research at Columbia University in New York. Madaule was searching for cDNA clones related to human peptide hormones (the α subunit of human chorionic gonadotropin) in the sea slug *Aplysia californica* and inadvertently came across the Rho gene through low stringency cloning [4]. A partial clone of the human RhoB gene was thus reported, and relatively soon the cloning of the genes of all three of the Rho proteins was accomplished: RhoA, RhoB, and RhoC [5,6]. The genes for additional Rho-like proteins were subsequently identified and cloned: Rac (Ras-related C3 botulinum toxin substrate) and Cdc42 (cell-division-cycle protein 42; also called G25K) [7,8,9,10]. There are two splice variants of Cdc42, known as placental [10] and brain [9] Cdc42, respectively.

The functions of the Rho proteins were also identified in a somewhat serendipitous manner, as work on *Clostridium botulinum* C3 toxin (an ADP ribosyl transferase) showed that it ADP ribosylated RhoC, a modification that rendered RhoC inactive [11,12]. Interestingly, C3 toxin treatment of Vero cells (from African green monkey kidneys) was shown to result in dramatic loss of filamentous actin, thereby providing the first hint that the Rho proteins might be involved in the regulation of actin dynamics [11]. Despite the original naming of Rac1 and Rac2 as C3 substrates, the toxin appeared to exclusively ADP ribosylate RhoA, RhoB, and RhoC on asparagine 41 [13,14]. This modification resulted in decreased signaling capacity of these Rho proteins, due to their stronger interactions with Rho GDP guanine nucleotide dissociation inhibitor (RhoGDI) and disruption of guanine nucleotide exchange factor (GEF)-dependent exchange of guanosine diphosphate (GDP) for guanosine triphosphate (GTP) [14]. The effects on actin filament organization were further confirmed in several seminal studies from the group of Alan Hall, which coined the concept of ‘archetypical’ Rho GTPases; i.e., the three members RhoA, Rac1, and Cdc42 [15,16,17,18]. According to the resulting paradigm, each of these had specific effects on cytoskeletal dynamics: activation of RhoA resulted in the formation of stress fibers; activation of Rac1 regulated lamellipodia formation; and activation of Cdc42 triggered formation of filopodia at the cell edges [19].

## 3. Some Basic Facts about Rho GTPases

The Rho GTPases belong to the Ras superfamily of small GTPases. This is a huge group of proteins that encompasses more than 150 members [20]. These proteins are guanine-nucleotide-binding enzymes that bind GTP and catalyze its hydrolysis to GDP. Studies over the last 30 years have shown that the majority of these Ras superfamily proteins follow a relatively simple scheme of activation: They have a different three-dimensional fold when they are in the GTP-bound conformation compared to when they are in the GDP-bound conformation. Most importantly, they are active in the GTP-bound state and inactive in the GDP-bound state [1]. Most of the changes in conformation occur in the structural elements known as Switch-I and Switch-II, which in Ras encompass amino-acid residues 30 to 38 and 60 to 76, respectively [21]. Although they function as enzymes, their catalytic activities are relatively low; however, the enzymatic activity can be stimulated at least 100-fold by the GTPase-activating proteins (GAPs) [22]. In addition, their exchange of GDP for GTP is facilitated by GEFs [23]. Thus, GAPs can be considered to be negative regulators of small GTPases, and GEFs as positive regulators.

There are roughly 70 different RhoGEFs and 80 different RhoGAPs, all of which have a unique spectra of affinities for the different Rho GTPases. Two types of mutations have been very useful in studies of Rho GTPases (as well as of other small GTPases), and their initial characterization actually arose from studies of tumor-associated mutations in the three Ras genes [23]. Mutations in position 12 (commonly, G12V) and 61 (in particular, Q61L) result in GTPase-deficient Rho proteins, which are therefore considered to be constitutively active. Rho GTPases harboring mutations in position 17 (e.g., Rac1/T17N) have reduced nucleotide binding, and this category of mutant Rho GTPases are considered to function in a dominant negative fashion because they sequester RhoGEFs and prevent them from functioning as exchange factors, thereby abrogate further signaling by Rho GTPases [24]. However, this model is probably not conclusive; many RhoGEFs have a broad spectrum of specificities and yet the codon 17 mutants of Rho, Rac, and Cdc42 are relatively specific for inhibition of the signaling of their respective Rho members (i.e., RhoA/T19N does not inhibit signaling by Cdc42).

Another important feature of small GTPases is their mode of posttranslational modification. The Ras GTPases are usually prenylated at the C-terminal so-called CAAX box, which is formed by a stretch of amino-acid residues at the C-terminus of the small GTPases (consensus sequence: Cysteine, followed by two aliphatic amino-acid residues, and a less defined ultimate amino-acid residue). For Ras, a 15-carbon farnesyl moiety is covalently attached to the cysteine, and subsequently the AAX peptide is removed and the cysteine becomes methylated [25]. This modification is required to confer targeting of Ras to lipid bilayers, such as the plasma membrane or endomembranes. In addition to the GEFs and GAPs, there is an additional family of Rho regulators, the Rho GDIs [26]. In humans, there are three RhoGDI members, and they bind to the prenylated Rho GTPases (Rho GTPases are usually geranyl-geranylated, rather than farnesylated) and sequester them in an inactive GDP-bound confirmation. Upon cell activation, the Rho GTPases dissociate from the RhoGDI complex and the released Rho GTPase can be activated by RhoGEFs.

**Figure 1:**
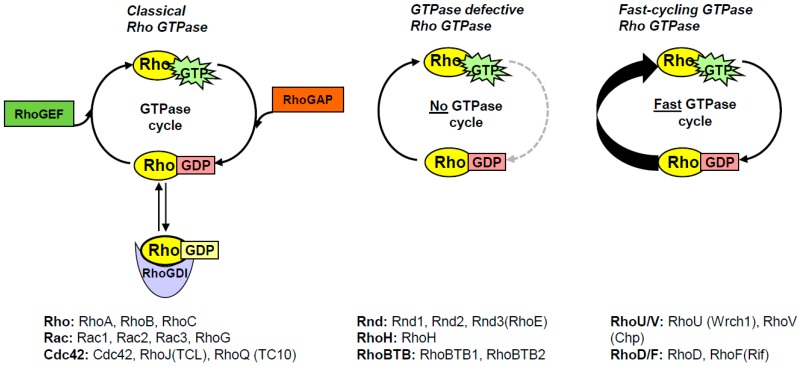
Schematic representation of the GTPase cycling of the different Rho GTPases. The classical Rho GTPases cycle between their GDP-bound and GTP-bound conformations, which is governed by RhoGDIs, RhoGEFs, and RhoGAPs. The Rho GTPases that belong to the respective categories are indicated below each panel.

As already mentioned, the Rho subfamily of small GTPases consists of 20 members that were all originally considered to follow the general GTP/GDP cycling regulated by GEFs and GAPs. However, it turns out that 10 of these Rho GTPases have other modes of activation, and are collectively known as atypical Rho GTPases [2]. They are still active when they are GTP-bound, but how they reach the active conformation differs between the different atypical Rho GTPases. In essence, there are two categories of atypical Rho GTPases, one that has a defective GTPase activity and one that has an increased intrinsic guanine nucleotide exchange activity (Figure 1). The GTPase defective Rho GTPases comprise Rnd1-3, RhoH, and RhoBTB1-2, which have alternative amino-acid residues in positions equivalent to 12, 59, and 61 (Ras numbering), and where mutations in the equivalent positions in Ras are known to result in constitutively GTP-bound small GTPases [23]. In addition, Rnd1-3 and RhoH are farnesylated rather than geranyl-geranylated, whereas RhoBTB1-2 do not have CAAX motifs, and it is not known if these proteins contain any membrane-targeting motifs [25,27]. The GTPase activity of theses atypical Rho GTPases cannot be stimulated by GAP-domain-containing proteins, although they appear to have intact GDP/GTP exchange activities [27,28]. RhoE is the best studied member of these GTPase-deficient Rho members, and it is likely that what has been shown in studies on RhoE is also applicable to the other Rnd members, and probably also to RhoH.

RhoE was shown to bind the RhoGEF Syx, although not through the DH/PH domain, but instead through a Ras-binding-domain–like motif in the N-terminus of Syx, which suggests that Syx is a RhoE effector rather than an activator [29]. RhoGDI does not appear to form an inhibitory complex with RhoE, while RhoGAP domains cannot stimulate the GTPase activity of RhoE, as the catalytically important so-called ‘arginine finger’ in the RhoGAP domain is sterically hindered, which prevents a functional interaction with RhoE [28]. RhoE can however bind p190RhoGAP, although the interaction occurs through a separate domain that is proximal to the C-terminal GAP domain and, similar to Syx, p190RhoGAP is most likely a RhoE effector rather than a regulator [30]. The RhoE/Rnd GTPases have been considered to function as RhoA antagonists because they were shown to cause stress fiber dissolution and cell rounding (hence the name Rnd) [31]. This inhibitory effect of Rnd GTPases on RhoA has been suggested to occur via p190RhoGAP, as at least RhoE increases the catalytic activity of the RhoGAP domain [30]. One remaining question here is how the GTPase-deficient Rho GTPases are inactivated. One possible mechanism is through their interactions with 14-3-3 proteins through a mechanism that involves phosphorylation of RhoE by ROCK and/or PKC, and the farnesyl moiety in the RhoE C-terminus. 14-3-3 proteins are known to bind target proteins through motifs that contain phosphorylated serine or threonine residues and thereby regulate the function of their interacting partners [32]. In the current situation, the 14-3-3 interaction result in exclusion of the phosphorylated RhoE from the plasma membrane (with Rnd1-2 apparently regulated in the same manner) [33].

RhoBTB1-2 are divergent from the other Rho GTPases, in terms of both their domain organization and their function [34]. It is not clear whether RhoBTB1-2 can bind nucleotides, in any respect, this function has not been fully resolved. There is a third member of the RhoBTBs, RhoBTB3, although the GTP-binding domain of RhoBTB3 is different from the Rho GTPases, and therefore RhoBTB3 is generally not included in the Rho family [35]. Interestingly, RhoBTB3 appears to function as an ATP hydrolyzing protein, albeit with a very slow ATPase activity [36]. Potentially, the nucleotide binding of the RhoBTB ‘GTPase’ domain is not essential for its function, and instead it is likely that it serves as a binding domain.

The RhoBTBs contain two broad complex–tramtrack–bric à brac (BTB) domains that are known to be involved in protein-protein interactions. In the RhoBTBs, these domains have been shown to mediate homodimerization or heterodimerization [37]. As opposed to the majority of the other Rho GTPases, the RhoBTBs have no obvious effects on the organization of the actin filament system [38]. Instead, they function as subunits in an E3 ubiquitin ligase complex together with Cullin 3 and the ring-finger domain protein ROC1 (Rbx1) [37,39]. In light of these characteristics, it is doubtful whether the RhoBTBs can be considered as members of the Rho GTPase family. Instead, it would appear to be more appropriate to treat RhoBTB1-3 as a separate subfamily of the Ras superfamily of small GTPases. If it is possible to find consensus for such a view, the Rho GTPase family of small GTPases would comprise 18 members.

Another class of atypical Rho GTPases includes RhoU, RhoV, RhoD, and RhoF. These atypical Rho GTPases are significantly different from the GTPase-deficient members, as they have elevated intrinsic nucleotide exchange activity; i.e., they do not need involvement of the GEFs to be activated [40,41]. Importantly, their hydrolysis activities are intact, which means that they can cycle between GDP-bound and GTP-bound conformations. This group of atypical Rho GTPases are referred to as the fast-cycling Rho GTPases [2]. The exchange of GDP for GTP is ensured in most cell types by the roughly 10-fold higher levels of GTP over GDP [42]. RhoU and RhoV were shown to be palmitoylated and not prenylated [43,44], while RhoD does not appear to be farnesylated or geranyl-geranylated, which is in contrast to RhoF, which can undergo both types of prenylation [25]. The fast-cycling Rho GTPases do not appear to bind RhoGDI, and to date, no GEFs or GAPs have been identified for this group of proteins. As the atypical Rho GTPases are constitutively active in their basic conformations, this suggests that they are regulated by other mechanisms, such as at the transcriptional level, or by targeted protein degradation [45].

Why are the behaviors of the classical Rho GTPases considered to be the ‘norm’, and the atypical members treated as the exceptions? In human cells, there are as many classical Rho GTPases as there are atypical Rho GTPases. I believe that the answer resides in the earlier identification of the classical members of Rho, Rac, and Cdc42, and their resemblance to the Ras proto-oncoproteins, their nucleotide binding characteristics, and their GTPase activities. However, there is one additional feature that makes the atypical Rho GTPases unique: They often contain additional domains or binding motifs. RhoU and RhoV have proline-rich extensions that can act as binding sites for SH3-domain-containing proteins, such as Nck, Grb2, and PLCγ [40,41,46]. The prevailing concept is that these N-terminal extensions have regulatory roles in the activation of RhoU and RhoV. An N-terminal deletion mutant of RhoU has increased affinity for Pak1, which is a downstream target of RhoU, and this deletion mutant induced increased kinase activity of Pak1 [41]. RhoD and RhoF also have N-terminal extensions, as 14 amino-acid residues in RhoD and 16 amino-acid residues in RhoF. The role of this extension in RhoF is not known, but in RhoD it appears to have a regulatory role, as deletion of the N-terminal extension resulted in relocalization of RhoD from EEA1-positive vesicles to CD63-positive vesicles [47].

## 4. Why Were the Rho GTPases Not Considered to Be Oncogenes?

Originally, the Rho GTPases were not classified as oncogenes, although a number of studies have reported that constitutively active mutants of RhoA and Rac1, and in particular RhoA/G14V and Rac1/G12V, can have transforming properties when tested in vitro in focus formation assays and transformation assays (e.g., for growth in soft agar). There were also indications that these active mutants of RhoA and Rac1 can cause tumor growth in nude mice [48,49,50]. The answer to this question is probably that despite their similarity to the Ras oncoproteins, no mutants in the Rho GTPases were identified in tumor samples or linked to human cancers. Instead, the predominant view was that Rho GTPases might be linked to cancer only through their cooperation with known oncogenes. For instance, it was shown that RhoA and Rac1 can cooperate with Ras and Raf in tumor progression [48,49].

Interestingly, some RhoGEF-domain-containing proteins were identified as oncogenes [23]. Dbl was the first protein of this type, and it was identified in diffuse B-cell lymphoma (hence the name Dbl) [51]. The oncogenic version of Dbl was shown to have its N-terminal 497 amino-acid residues deleted, out of the 925 amino-acids residues of full-length Dbl, with the deletion replaced with a sequence from an unrelated protein [52]. A number of additional RhoGEF proteins have been shown to be activated by N-terminal truncations, most notably Ect2 and Vav1-3 [23]. This indicated that constitutive triggering of the GDP/GTP cycling of Rho GTPases caused cell transformation. From these observations it became apparent that the Rho GTPases could be turned into oncoproteins if their intrinsic exchange activity was under constant triggering by a constitutively active RhoGEF. So, how are the RhoGEF proteins activated under normal circumstances? Truncation is clearly an activating cue, although it does not appear to be likely to be the common mechanism in vivo. Release of an autoinhibited conformation is a more plausible mechanism. In line with this concept, intramolecular interactions have been shown to negatively affect the GEF activity of several RhoGEF proteins [23,53]. In addition, there have been numerous indications that RhoGEF proteins are up-regulated in tumors, although it is not clear if this is an event that drives cancer progression. We obviously need more information regarding how dysregulated functions of RhoGEFs might cause cancers.

DOCK-domain proteins are another category of proteins with Rho GEF activities. In humans, there are 11 members of these proteins, named DOCK1 to DOCK11 [54,55]. No oncogenic mutations in the DOCK proteins have been described to date, although several members are known to have important roles in regulation of cancer-cell migration and invasion (for further details, see [55]). What about mutations in RhoGDIs or Rho GAPs? RhoGDIs have been correlated to the regulation of cancer-cell migration, but how this is achieved is currently relatively vague [26,56]. RhoGDI does not bind to the fast-cycling Rho GTPases, most likely because they have no functional CAAX boxes, and therefore they do not undergo posttranslational modifications by geranyl-geranylation or farnesylation. Theoretically, RhoGAP-domain-containing proteins can be expected to be down-regulated, to thereby extend the time spent by the Rho GTPases in their GTP-bound conformations. If this hypothesis holds true, RhoGAPs might serve as tumor suppressors. Indeed, one group of RhoGAP proteins, as ‘deleted in liver cancer’ (DLC), was identified as tumor suppressors [57]. In humans, there are three DLC paralogs, and each comes in a variety of splice variants. DLCs are commonly expressed in a variety of human cancers, as well as in cancer cell lines [58,59]. In addition, there are reports of decreased expression of certain RhoGAPs in cancers, although more work is required to determine whether other RhoGAPs can serve as tumor suppressors [60].

## 5. An Oncogenic Splice Variant of Rac1

The first mutant Rho member that was linked to cancer was a splice variant of Rac1 that is known as Rac1B. This variant arises because of an alternative splicing event that provides 19 extra amino-acid residues following amino-acid residue 75, immediately after the Switch-II motif; this results in a protein of 211 amino-acid residues rather than the normal 192 [61,62]. Importantly, the Rac1B variant turned out to have a much higher intrinsic GDP/GTP exchange activity and might therefore qualify as a fast-cycling mutant protein [62,63]. The exon giving rise to the alternative splicing can be detected exclusively in amniotes; however, it is not known if this also implies that the Rac1b splice variant will occur in other species [35]. It is not clear why this splice form has been conserved, but it implies that Rac1b has been positively selected for a physiological function, which would appear to be related to cell adhesion. Rac1b is not only associated to cancers in terms of its increased expression, the mutant protein can also promote cell transformation in the classical focus forming assay [64].

Which signaling pathway involves Rac1b in cancer? Rac1b appears to function differently from the normal splice variant of Rac1. For instance, Rac1b does not bind RhoGDI, so this regulatory protein cannot sequester Rac1b in an inactive complex [64]. Moreover, Rac1b has a lower affinity for a number of common Rac1 effectors, such as Pak1, IQGAP1, GIT1, and RACK1 (Table 1) [65]. Interestingly, Rac1b was shown to bind the adhesion protein 120 catenin through its extra 19 amino-acid residues [65]. The mode of action of Rac1b in cancers is not known, although one possible mode of action is that Rac1b can antagonize the activity of wild-type Rac1 [66,67]. Alternatively, the constitutively active nature of Rac1b results in dysregulation of the Rac1 signal, which then simply overrides the normal Rac1 protein.

## 6. The Concept of Fast-Cycling Small GTPases

The concept behind the fast-cycling small GTPases originates from studies in the early 1990s on the three-dimensional structure of H-Ras. This involved the search for detailed information regarding the amino-acid residues in H-Ras that were necessary for its interaction with the guanosine of GTP. To investigate this, a number of site-specific mutants were created, and the mutants with a phenylalanine substituted by a leucine at codon 28 (H-Ras/F28L) showed an increased nucleotide dissociation rate; this, in turn, resulted in higher intrinsic GDP/GTP exchange activity [71]. Interestingly, the expression of H-Ras/F28L in PC12 cells promoted neurite outgrowth, which indicated that H-Ras/F28L acts as a constitutively active H-Ras mutant.

The link between fast-cycling mutant small GTPases and oncogenic transformation was further substantiated in studies on the F28L mutants of Cdc42 and Rac2. These mutant Rho GTPases were also shown to have elevated intrinsic GDP/GTP exchange rates, in line with the H-Ras/F28L mutant [72,73]. Cdc42/F28L (Rac2/F28L was not tested) turned out to have oncogenic properties, as it promoted anchorage-independent growth of NIH3T3 cells in soft agar [72]. The Phe28 resides in the nucleotide-binding pocked of H-Ras, Rac1, and Cdc42, and it is particularly involved in their binding to the guanine base of GTP through a hydrophobic interaction. This interaction is lost in the F28L mutant, which explains why these F28L mutants had increased GDP/GTP exchange activities [74,75,76].

**Figure 2:**
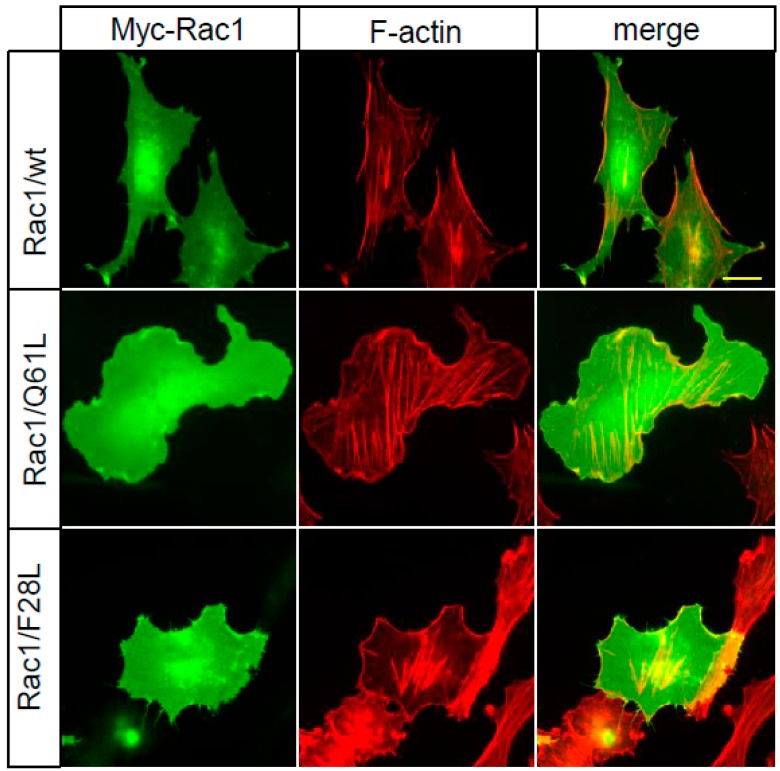
Rac1/F28L has a different effect on actin filament organization and cell morphology compared to Rac1/Q61L. Porcine aortic endothelial cells were transfected with Myc-constructs expressing Rac1/wt, Rac1/Q61L or Rac1/F28L. Myc-tagged Rac1 was visualized using a mouse anti-Myc antibody, followed by an Alexa Fluor 488-conjugated anti-mouse antibody. Filamentous actin was visualized with TRITC-conjugated phalloidin. Scale bar, 20 µm.

Why are the atypical Rho GTPases RhoD, RhoF, RhoU, and RhoV fast-cycling? The exact mechanism is not known, although RhoU and RhoV have tyrosines instead of phenylalanine residues in the positions equivalent to codon 28 in Rac1, and this is likely to reduce the hydrophobic interactions with the guanine base. However, it is not clear if this difference is sufficient to render the GTPases fast-cycling. In similarity to the classical Rho GTPases, RhoD and RhoF have phenylalanines in the position corresponding to codon 28 in Rac1, but the amino-acid sequence surrounding this position is relatively divergent from Rac1, RhoA, and Cdc42, which is likely to affect the nucleotide binding properties (for further discussion, see [77]). It is clear that both fast-cycling atypical Rho GTPases and mutants of the classical Rho GTPases should be considered as *bona-fide* constitutively active mutants. However, the mechanisms are different from the constitutively active mutants of the small GTPases that are in much wider use, such as the G12V and Q61L mutants [78]. These proteins have defective GTPase activities, but have retained their nucleotide exchange capacity. Importantly, the biological outcomes of the expression of Rac1/F28L and Rac1/Q61L are different. As an example of this, when Rac1/Q61L was expressed in porcine endothelial cells, this resulted in the formation of broad lamellae, whereas Rac1/F28L resulted in micro spikes, or short filopodia, at the cell periphery (Figure 2).

## 7. Cancer-Associated Point Mutations in Rac1

A number of genome-wide studies have resulted in what we can now see as the dawn of a new paradigm for the Rho GTPases and cancers. A relatively extensive mutation analysis of sun-exposed melanomas resulted in the identification of a novel point mutation in Rac1, as Rac1/P29S (Table 2) [79]. Studies of melanomas have shown that Rac1/P29S is found in about 5% to 9% of all melanomas. Interestingly, Rac1/P29S was shown to be the third most recurrent mutation in melanomas, after the B-Raf/V600 and N-Ras/Q61 mutations, and it is considerably more frequent in samples that were wild-type for B-Raf or N-Ras [79]. Rac1/P29S has a significantly increased intrinsic GDP/GTP cycling activity, although the mechanism underlying the fast cycling of this mutant is likely to be different from that of Rac1/F28L. The three-dimensional structure of Rac1/P29S in the presence of nonhydrolyzable GTP analogs has shown significant differences in the folding of the Switch-I motif, and this Rac1/P29S mutant shows a Switch-I conformation that more resembles that of Ras [76]. Rac1/P29S has increased affinity for the effectors Pak1 and MLK3, which is different from Rac1b, which appears to have lost its binding to many effectors [65].

Since the identification of Rac1/P29S in melanomas, there have been additional reports of Rac1 and Rac2 mutants, as identified in public databases and in common cell lines. For example, in HT1080 cells, a new fast-cycling mutant of Rac1 was identified, Rac1/N92I, but in general, the biological functions of these mutations are not so clear [81,87]. It is also not so clear what the targets of Rac1/P29S are. The expression of PD-L1 was shown to be regulated by Rac1/P29S but not by other Rac1 mutants [88]. Expression of Rac1/P29S has also been shown to abrogate haptotaxis in fibroblasts (i.e., direct cell migration triggered by increased concentrations of extracellular matrix proteins, here fibronectin) [89]. Not only was the directed cell migration affected, but also the velocity was decreased, although not the persistence. Another study showed that Rac1/P29S expression inhibited invadopodia formation in melanoma cell lines [90]. These observations show that the mechanism behind Rac1-GTP/P29S in cancer progression is complex and does not only involve increased formation of Rac1-GTP.

## 8. Cancer-Associated Point Mutations in Other Rho GTPases

Recurrent somatic mutations in the *RHOA* gene were recently shown for patients suffering from angioimmunoblastic T-cell lymphoma using whole-exome sequencing (Table 2) [83]. The most common mutation turned out to be G17V in RhoA, which is not a fast-cycling activated mutant. Instead, it appears to act as a dominant-negative RhoA protein, similar to the conventional (and artificial) RhoA/T19N mutant. Another whole genome sequencing effort of patients with adult T-cell leukemia/lymphoma resulted in the identification of a number of novel point mutations in the RhoA gene in 15% of the tumor material [85]. Many of these mutations were caused by hits to the same site. The modes of action of dominant-negative mutants of RhoA in cancers are not clear, but as there is an inverse relationship between RhoA signaling and Rac1 signaling, at least in some cell types it is possible that inhibition of the Rho-regulated pathway results in increased Rac1 signaling [91].

Recently, somatic mutation in Cdc42 was also reported, this time in well-differentiated papillary mesothelioma [86]. Three mutations in Cdc42 were described: Two Q61R mutations and one P34Q mutation. The nucleotide binding characteristics of these mutants were not evaluated, but mutation of amino acid 61 has been shown to result in GTPases-deficient proteins, and the proline in position 34 resides in the effector-binding loop and can be envisioned to affect the binding of Cdc42 to its downstream effectors [18].

## 9. Summary

The recent decades have seen a shift in the paradigm, and the field of Rho GTPases has clearly moved into the heartland of cancer biology. The identification of cancer-associated somatic mutations in Rac1 and RhoA, and more recently also in Cdc42, has demonstrated that the Rho GTPases appear to have more active roles in cancer progression than originally thought. However, there remains a lot more to be learnt about the roles of the mutant proteins in tumor progression. We do not understand how dominant-negative mutants of RhoA can cause cancers, and we do not understand why Rac1/P29S can be an oncogene when it negatively regulates adhesion-dependent cell migration and invadopodia formation. These observations appear counterintuitive in a cancer setting. Looking in the databases, such as the COSMIC (Catalogue Of Somatic Mutations In Cancer) database, it is clear that there are several additional mutations in the genes that encode the Rho GTPases, and we certainly need to learn more about the complex signaling networks that involve the Rho GTPases. It is likely that Rho GTPases and their downstream pathways can serve as druggable targets for cancer treatments, either alone or in combination with other targets, such as the Ras/B-Raf pathway.

## Figures and Tables

**Table 1:** Proteins that are known to bind or not to bind to Rac1b.

Proteins Known to Bind to Rac1b		Rac1-Binding Proteins that *Do Not* Bind Rac1b	
Protein	Reference	Protein	Reference
smgGDS	[65]	Pak1	[63,64]
RACK1		RhoGDI	[63,64,65,66]
p120 ^ctn^		IQGAP1	[65]
ROCK1 *	[68]	GIT-1	
Cytochrome C *			
NADPH oxidase *	[69]		
Dishevelled-3 *	[70]		
β-Catenin *			
TCF-4 *			

* It is not clear if these proteins also bind Rac1/wt.

**Table 2:** Known cancer-associated mutants of Rac1, Rac2, RhoA, and Cdc42.

Small GTPase	Mutant	Cell Type	Reference
Rac1	P29S	Sun-exposed melanoma	[79]
		Melanoma	[80]
		Mouth, squamous cell carcinoma	[81]
		MDA-MB-157 cell line	
	N92I	HT1080 fibrosarcoma cell line	[81]
	C157Y	Lung, adenocarcinoma	
	P179L	Skin, squamous cell carcinoma	
Rac2	P29L	Melanoma	[80]
	I21M	Larynx, squamous cell carcinoma	[81]
	P29L	HCC1143 cell line	
	P29Q	KCL-22 cell line	
	D47Y	Glioma	
	P106H	Malignant melanoma	
	D63V	Juvenile myelomonocytic leukemia	[82]
RhoA	G17V	Angioimmunoblastic T cell lymphoma	[83]
	A161E		
	R5Q	Angioimmunoblastic T cell lymphoma	[84]
	G17V	Adult T-cell leukemia/lymphoma	[85]
	G14V		
	C16R		
	C16F		
	C16G		
	C16L		
	T19I		
	A56V		
	R68L		
	C83Y		
	N117I		
	K118E		
	K118Q		
	D120N		
	A161P		
	A161V		
	K162E		
Cdc42	Cdc42/Q61R	Papillary mesothelioma	[86]
	Cdc42/P34Q		
	Cdc42/G12D	Melanoma	[80]

## References

[1] Wennerberg K., Der C.J. (2004). Rho-family GTPases: It’s not only Rac and Rho (and I like it). J. Cell Sci..

[2] Aspenström P. (2018). Fast-cycling Rho GTPases. Small GTPases.

[3] Bustelo X.R. (2018). RHO GTPases in cancer: Known facts, open questions, and therapeutic challenges. Biochem. Soc. Trans..

[4] Madaule P., Axel R. (1985). A novel Ras-related gene family. Cell.

[5] Yeramian P., Chardin P., Madaule P., Tavitian A. (1987). Nucleotide sequence of human rho cDNA clone 12. Nucl. Acids Res..

[6] Chardin P., Madaule P., Tavitian A. (1988). Coding sequence of human rho cDNAs clone 6 and clone 9. Nucl. Acids Res..

[7] Evans T., Brown M.L., Fraser E.D., Northup J.K. (1986). Purification of the major GTP-binding proteins from human placental membranes. J. Biol. Chem..

[8] Didsbury J., Weber R.F., Bokoch G.M., Evans T., Snyderman R. (1989). Rac, A novel ras-related family of proteins that are botulinum toxin substrates. J. Biol. Chem..

[9] Munemitsu S., Innis M.A., Clark R., McCormick F., Ullrich A., Polakis P. (1990). Molecular cloning and expression of a G25K cDNA, the human homolog of the yeast cell cyclegene CDC42. Mol. Cell. Biol..

[10] Shinjo K., Koland J.G., Hart M.J., Narasimhan V., Johnson D.I., Evans T., Cerione R.A. (1990). Molecular cloning of the gene for the human placental GTP-binding protein Gp (G25K): Identification of this GTP-binding protein as the human homolog of the yeast cell-division-cycle protein CDC42. Proc. Natl. Acad. Sci. USA.

[11] Chardin P., Boquet P., Madaule P., Popoff M.R., Rubin E.J., Gill D.M. (1989). The mammalian G protein rhoC is ADP-ribosylated by *Clostridium botulinum* exoenzyme C3 and affects actin microfilaments in Vero cells. EMBO J..

[12] Braun U., Habermann B., Just I., Aktories K., Vandekerckhove J. (1989). Purification of the 22 kDa protein substrate of botulinum ADP-ribosyltransferase C3 from porcine brain cytosol and its characterization as a GTP-binding protein highly homologous to the rho gene product. FEBS Lett..

[13] Nemoto Y., Namba T., Teru-uchi T., Ushikubi F., Morii N., Narumiya S. (1992). A rho gene product in human blood platelets. I. Identification of the platelet substrate for botulinum C3 ADP-ribosyltransferase as rhoA protein. J. Biol. Chem..

[14] Aktories K. (2015). Rho-modifying bacterial protein toxins. Pathog. Dis..

[15] Paterson H.F., Self A.J., Garrett M.D., Just I., Aktories K., Hall A. (1990). Microinjection of recombinant p21rho induces rapid changes in cell morphology. J. Cell Biol..

[16] Ridley A.J., Hall A. (1992). The small GTP-binding protein rho regulates the assembly of focal adhesions and actin stress fibers in response to growth factors. Cell.

[17] Ridley A.J., Paterson H.F., Johnston C.L., Diekmann D., Hall A. (1992). The small GTP-binding protein rac regulates growth factor-induced membrane ruffling. Cell.

[18] Nobes C.D., Hall A. (1995). Rho, rac, and cdc42 GTPases regulate the assembly of multimolecular focal complexes associated with actin stress fibers, lamellipodia, and filopodia. Cell.

[19] Hall A. (2012). Rho family GTPases. Biochem. Soc. Trans..

[20] Colicelli J. (2004). Human RAS superfamily proteins and related GTPases. Sci. STKE.

[21] Cox A.D., Der C.J. (2010). Ras history: The saga continues. Small GTPases.

[22] Tcherkezian J., Lamarche-Vane N. (2007). Current knowledge of the large RhoGAP family of proteins. Biol. Cell.

[23] Cook D.R., Rossman K.L., Der C.J. (2014). Rho guanine nucleotide exchange factors: Regulators of Rho GTPase activity in development and disease. Oncogene.

[24] Feig L.A. (1999). Tools of the trade: Use of dominant-inhibitory mutants of Ras-family GTPases. Nat. Cell Biol..

[25] Roberts P.J., Mitin N., Keller P.J., Chenette E.J., Madigan J.P., Currin R.O., Cox A.D., Wilson O., Kirschmeier P., Der C.J. (2008). Rho family GTPase modification and dependence on CAAX motif-signaled posttranslational modification. J. Biol. Chem..

[26] Xie F., Shao S., Aziz A.U.R., Zhang B., Wang H., Liu B. (2017). Role of Rho-specific guanine nucleotide dissociation inhibitor α regulation in cell migration. Acta Histochem..

[27] Foster R., Hu K.Q., Lu Y., Nolan K.M., Thissen J., Settleman J. (1996). Identification of a novel human Rho protein with unusual properties: GTPase deficiency and in-vivo farnesylation. Mol. Cell. Biol..

[28] Fiegen D., Blumenstein L., Stege P., Vetter I.R., Ahmadian M.R. (2002). Crystal structure of Rnd3/RhoE: Functional implications. FEBS Lett..

[29] Goh L.L., Manser E. (2010). The RhoA GEF Syx is a target of Rnd3 and regulated via a Raf1-like ubiquitin-related domain. PLoS ONE.

[30] Wennerberg K., Forget M.A., Ellerbroek S.M., Arthur W.T., Burridge K., Settleman J., Der C.J., Hansen S.H. (2003). Rnd proteins function as RhoA antagonists by activating p190 RhoGAP. Curr. Biol..

[31] Nobes C.D., Lauritzen I., Mattei M.G., Paris S., Hall A., Chardin P. (1998). A new member of the Rho family, Rnd1, promotes disassembly of actin filament structures and loss of cell adhesion. J. Cell Biol..

[32] Brandwein D., Wang Z. (2017). Interaction between Rho GTPases and 14-3-3 Proteins. Int. J. Mol. Sci..

[33] Riou P., Kjær S., Garg R., Purkiss A., George R., Cain R.J., Bineva G., Reymond N., McColl B., Thompson A.J. (2013). 14-3-3 proteins interact with a hybrid prenyl-phosphorylation motif to inhibit G proteins. Cell.

[34] Ji W., Rivero F. (2016). Atypical Rho GTPases of the RhoBTB subfamily: Roles in vesicle trafficking and tumorigenesis. Cells.

[35] Boureux A., Vignal E., Faure S., Fort P. (2007). Evolution of the Rho family of Ras-like GTPases in eukaryotes. Mol. Biol. Evol..

[36] Espinosa E.J., Calero M., Sridevi K., Pfeffer S.R. (2009). RhoBTB3: A Rho GTPase-family ATPase required for endosome to Golgi transport. Cell.

[37] Berthold J., Schenková K., Ramos S., Miura Y., Furukawa M., Aspenström P., Rivero F. (2008). Characterization of RhoBTB-dependent Cul3 ubiquitin ligase complexes—Evidence for an autoregulatory mechanism. Exp. Cell Res..

[38] Aspenström P., Fransson A., Saras J. (2004). Rho GTPases have diverse effects on the organization of the actin filament system. Biochem. J..

[39] Wilkins A., Ping Q., Carpenter C.L. (2004). RhoBTB2 is a substrate of the mammalian Cul_3_ ubiquitin ligase complex. Genes Dev..

[40] Saras J., Wollberg P., Aspenström P. (2004). Wrch1 is a GTPase-deficient Cdc42-like protein with unusual binding characteristics and cellular effects. Exp. Cell Res..

[41] Shutes A., Berzat A.C., Cox A.D., Der C.J. (2004). Atypical mechanism of regulation of the Wrch-1 Rho family small GTPase. Curr. Biol..

[42] Traut T.W. (1994). Physiological concentrations of purines and pyrimidines. Mol. Cell Biochem..

[43] Berzat A.C., Buss J.E., Chenette E.J., Weinbaum C.A., Shutes A., Der C.J., Minden A., Cox A.D. (2005). Transforming activity of the Rho family GTPase, Wrch-1, a Wnt-regulated Cdc42 homolog, is dependent on a novel carboxyl-terminal palmitoylation motif. J. Biol. Chem..

[44] Chenette E.J., Abo A., Der C.J. (2005). Critical and distinct roles of amino- and carboxyl-terminal sequences in regulation of the biological activity of the Chp atypical Rho GTPase. J. Biol. Chem..

[45] Chardin P. (2006). Function and regulation of Rnd proteins. Nat. Rev. Mol. Cell Biol..

[46] Risse S.L., Vaz B., Burton M.F., Aspenström P., Piekorz R.P., Brunsveld L., Ahmadian M.R. (2013). SH3-mediated targeting of Wrch1/RhoU by multiple adaptor proteins. Biol. Chem..

[47] Blom M., Reis K., Aspenström P. (2018). RhoD localization and function is dependent on its GTP/GDP-bound state and unique N-terminal motif. Eur. J. Cell Biol..

[48] Qiu R.G., Chen J., McCormick F., Symons M. (1995). A role for Rho in Ras transformation. Proc. Natl. Acad. Sci. USA.

[49] Khosravi-Far R., Solski P.A., Clark G.J., Kinch M.S., Der C.J. (1995). Activation of Rac1, RhoA, and mitogen-activated protein kinases is required for Ras transformation. Mol. Cell. Biol..

[50] Del Peso L., Hernández-Alcoceba R., Embade N., Carnero A., Esteve P., Paje C., Lacal J.C. (1997). Rho proteins induce metastatic properties in vivo. Oncogene.

[51] Eva A., Aaronson S.A. (1985). Isolation of a new human oncogene from a diffuse B-cell lymphoma. Nature.

[52] Ron D., Tronick S.R., Aaronson S.A., Eva A. (1988). Molecular cloning and characterization of the human dbl proto-oncogene: Evidence that its overexpression is sufficient to transform NIH/3T3 cells. EMBO J..

[53] Raimondi F., Felline A., Fanelli F. (2015). Catching functional modes and structural communication in Dbl family Rho guanine nucleotide exchange factors. J. Chem. Inf. Model..

[54] Côté J.F., Vuori K. (2002). Identification of an evolutionarily conserved superfamily of DOCK180-related proteins with guanine nucleotide exchange activity. J. Cell Sci..

[55] Gadea G., Blangy A. (2014). Dock-family exchange factors in cell migration and disease. Eur. J. Cell Biol..

[56] Harding M.A., Theodorescu D. (2010). RhoGDI signaling provides targets for cancer therapy. Eur. J. Cancer.

[57] Yuan B.Z., Miller M.J., Keck C.L., Zimonjic D.B., Thorgeirsson S.S., Popescu N.C. (1998). Cloning, characterization, and chromosomal localization of a gene frequently deleted in human liver cancer (DLC-1) homologous to rat RhoGAP. Cancer Res..

[58] Braun A.C., Olayioye M.A. (2015). Rho regulation: DLC proteins in space and time. Cell Signal..

[59] Lukasik D., Wilczek E., Wasiutynski A., Gornicka B. (2011). Deleted in liver cancer protein family in human malignancies. Oncol. Lett..

[60] Vigil D., Cherfils J., Rossman K.L., Der C.J. (2010). Ras superfamily GEFs and GAPs: Validated and tractable targets for cancer therapy?. Nat. Rev. Cancer.

[61] Jordan P., Brazåo R., Boavida M.G., Gespach C., Chastre E. (1999). Cloning of a novel human Rac1b splice variant with increased expression in colorectal tumors. Oncogene.

[62] Schnelzer A., Prechtel D., Knaus U., Dehne K., Gerhard M., Graeff H., Harbeck N., Schmitt M., Lengyel E. (2000). Rac1 in human breast cancer: Overexpression, mutation analysis, and characterization of a new isoform, Rac1b. Oncogene.

[63] Fiegen D., Haeusler L.C., Blumenstein L., Herbrand U., Dvorsky R., Vetter I.R., Ahmadian M.R. (2004). Alternative splicing of Rac1 generates Rac1b, a self-activating GTPase. J. Biol. Chem..

[64] Singh A., Karnoub A.E., Palmby T.R., Lengyel E., Sondek J., Der C.J. (2004). Rac1b, a tumor associated, constitutively active Rac1 splice variant, promotes cellular transformation. Oncogene.

[65] Orlichenko L., Geyer R., Yanagisawa M., Khauv D., Radisky E.S., Anastasiadis P.Z., Radisky D.C. (2010). The 19-amino acid insertion in the tumor-associated splice isoform Rac1b confers specific binding to p120 catenin. J. Biol. Chem..

[66] Matos P., Collard J.G., Jordan P. (2003). Tumor-related alternatively spliced Rac1b is not regulated by Rho-GDP dissociation inhibitors and exhibits selective downstream signaling. J. Biol. Chem..

[67] Nimnual A.S., Taylor L.J., Nyako M., Jeng H.H., Bar-Sagi D. (2010). Perturbation of cytoskeleton dynamics by the opposing effects of Rac1 and Rac1b. Small GTPases.

[68] Kang H.T., Park J.T., Choi K., Choi H.J.C., Jung C.W., Kim G.R., Lee Y.S., Park S.C. (2017). Chemical screening identifies ROCK as a target for recovering mitochondrial function in Hutchinson-Gilford progeria syndrome. Aging Cell.

[69] Lee K., Chen Q.K., Lui C., Cichon M.A., Radisky D.C., Nelson C.M. (2012). Matrix compliance regulates Rac1b localization, NADPH oxidase assembly, and epithelial-mesenchymal transition. Mol. Biol. Cell.

[70] Pethe V.V., Charames G.S., Bapat B. (2011). Rac1b recruits Dishevelled and β-catenin to Wnt target gene promoters independent of Wnt3A stimulation. Int. J. Oncol..

[71] Reinstein J., Schlichting I., Frech M., Goody R.S., Wittinghofer A. (1991). P21 with a phenylalanine 28→leucine mutation reacts normally with the GTPase-activating protein GAP, but nevertheless has transforming properties. J. Biol. Chem..

[72] Lin R., Bagrodia S., Cerione R., Manor D. (1997). A novel Cdc42Hs mutant induces cellular transformation. Curr. Biol..

[73] Xu X., Wang Y., Barry D.C., Chanock S.J., Bokoch G.M. (1997). Guanine nucleotide binding properties of Rac2 mutant proteins and analysis of the responsiveness to guanine nucleotide dissociation stimulator. Biochemistry.

[74] Schlichting I., John J., Frech M., Chardin P., Wittinghofer A., Zimmermann H., Rösch P. (1990). Proton NMR studies of transforming and nontransforming H-ras p21 mutants. Biochemistry.

[75] Adams P.D., Oswald R.E. (2006). Solution structure of an oncogenic mutant of Cdc42Hs. Biochemistry.

[76] Davis M.J., Ha B.H., Holman E.C., Halaban R., Schlessinger J., Boggon T.J. (2013). RAC1P29S is a spontaneously activating cancer-associated GTPase. Proc. Natl. Acad. Sci. USA.

[77] Jaiswal M., Fansa E.K., Dvorsky R., Ahmadian M.R. (2013). New insight into the molecular switch mechanism of human Rho family proteins: Shifting a paradigm. Biol. Chem..

[78] Krengel U., Schlichting I., Scherer A., Schumann R., Frech M., John J., Kabsch W., Pai E.F., Wittinghofer A. (1990). Three-dimensional structures of H-ras p21 mutants: Molecular basis for their inability to function as signal switch molecules. Cell.

[79] Krauthammer M., Kong Y., Ha B.H., Evans P., Bacchiocchi A., McCusker J.P., Cheng E., Davis M.J., Goh G., Choi M. (2012). Exome sequencing identifies recurrent somatic RAC1 mutations in melanoma. Nat. Genet..

[80] Hodis E., Watson I.R., Kryukov G.V., Arold S.T., Imielinski M., Theurillat J.P., Nickerson E., Auclair D., Li L., Place C. (2012). A landscape of driver mutations in melanoma. Cell.

[81] Kawazu M., Ueno T., Kontani K., Ogita Y., Ando M., Fukumura K., Yamato A., Soda M., Takeuchi K., Miki Y. (2013). Transforming mutations of RAC guanosine triphosphatases in human cancers. Proc. Natl. Acad. Sci. USA.

[82] Caye A., Strullu M., Guidez F., Cassinat B., Gazal S., Fenneteau O., Lainey E., Nouri K., Nakhaei-Rad S., Dvorsky R. (2015). Juvenile myelomonocytic leukemia displays mutations in components of the Ras pathway and the PRC2 network. Nat. Genet..

[83] Sakata-Yanagimoto M., Enami T., Yoshida K., Shiraishi Y., Ishii R., Miyake Y., Muto H., Tsuyama N., Sato-Otsubo A., Okuno Y. (2014). Somatic RHOA mutation in angioimmunoblastic T cell lymphoma. Nat. Genet..

[84] Yoo H.Y., Sung M.K., Lee S.H., Kim S., Lee H., Park S., Kim S.C., Lee B., Rho K., Lee J.E. (2014). A recurrent inactivating mutation in RHOA GTPase in angioimmunoblastic T cell lymphoma. Nat Genet..

[85] Nagata Y., Kontani K., Enami T., Kataoka K., Ishii R., Totoki Y., Kataoka T.R., Hirata M., Aoki K., Nakano K. (2016). Variegated RHOA mutations in adult T-cell leukemia/lymphoma. Blood.

[86] Stevers M., Rabban J.T., Garg K., Van Ziffle J., Onodera C., Grenert J.P., Yeh I., Bastian B.C., Zaloudek C., Solomon D.A. (2018). Well-differentiated papillary mesothelioma of the peritoneum is genetically defined by mutually exclusive mutations in TRAF7 and CDC42. Mod. Pathol..

[87] Mar V.J., Wong S.Q., Logan A., Nguyen T., Cebon J., Kelly J.W., Wolfe R., Dobrovic A., McLean C., McArthur G.A. (2014). Clinical and pathological associations of the activating RAC1 P29S mutation in primary cutaneous melanoma. Pigment Cell Melanoma Res..

[88] Vu H.L., Rosenbaum S., Purwin T.J., Davies M.A., Aplin A.E. (2015). Rac1 P29S regulates PD-L1 expression in melanoma. Pigment Cell Melanoma Res..

[89] King S.J., Asokan S.B., Haynes E.M., Zimmerman S.P., Rotty J.D., Alb J.G., Tagliatela A., Blake D.R., Lebedeva I.P., Marston D. (2016). Lamellipodia are crucial for haptotactic sensing and response. J. Cell Sci..

[90] Revach O.Y., Winograd-Katz S.E., Samuels Y., Geiger B. (2016). The involvement of mutant Rac1 in the formation of invadopodia in cultured melanoma cells. Exp. Cell Res..

[91] Sanz-Moreno V., Gadea G., Ahn J., Paterson H., Marra P., Pinner S., Sahai E., Marshall C.J. (2008). Rac activation and inactivation control plasticity of tumor cell movement. Cell.

